# Genome-Wide Microarray Analysis Suggests Transcriptomic Response May Not Play a Major Role in High- to Low-Altitude Acclimation in Harvest Mouse (*Micromys minutus*)

**DOI:** 10.3390/ani9030092

**Published:** 2019-03-13

**Authors:** Tze-Ho Chen, Gwo-Chin Ma, Wen-Hsiang Lin, Dong-Jay Lee, Sheng-Hai Wu, Ben-Yang Liao, Ming Chen, Liang-Kong Lin

**Affiliations:** 1 Department of Life Science, Tunghai University, Taichung 40704, Taiwan; 46305@cch.org.tw; 2 Department of Obstetrics and Gynecology, Changhua Christian Hospital, Changhua 50006, Taiwan; 3 Department of Genomic Medicine and Center for Medical Genetics, Changhua Christian Hospital, Changhua 50046, Taiwan; 128729@cch.org.tw (G.-C.M.); 397620cch@gmail.com (W.-H.L.); 118862@cch.org.tw (D.-J.L.); 4 Department of Genomic Science and Technology, Changhua Christian Hospital Healthcare System, Changhua Christian Hospital, Changhua 50046, Taiwan; 5 Department of Medical Laboratory Science and Biotechnology, Central Taiwan University of Science and Technology, Taichung 40601, Taiwan; 6 Department of Life Sciences, National Chung Hsing University, Taichung 40227, Taiwan; shwu@dragon.nchu.edu.tw; 7 Division of Biostatistics & Bioinformatics, Institute of Population Health Sciences, National Health Research Institutes, Zhunan, Miaoli County 35053, Taiwan; liaoby@nhri.org.tw; 8 Department of Obstetrics and Gynecology, College of Medicine, National Taiwan University, Taipei 10041, Taiwan; 9 Department of Medical Genetics, National Taiwan University Hospital, Taipei 10041, Taiwan; 10 Department of Molecular Biotechnology, Da-Yeh University, Changhua 51591, Taiwan

**Keywords:** gene expression, cDNA, acclimation, microarray, functional genomics, behavior

## Abstract

**Simple Summary:**

*Micromys minutus* is a small rodent species that has a wide range of vertical distribution in Taiwan. By comparing the gene expression profile of the skeletal muscle tissues taken from individuals native to the high-altitude environment and those transferred to the low-altitude captive site, the *Tnfrsf12a* gene was demonstrated to have a differential expression pattern. Although this finding may be correlated with the altitude acclimation, the observation of only one gene transcript with significant alteration leads us to suggest that genetic response may not play a major role in altitude acclimation in *M. minutus*. Future comparative functional genomics studies involving other organ systems (in addition to skeletal muscles) and alarger sample size are warranted for better insight into the altitude acclimation of this small rodent species.

**Abstract:**

The harvest mouse (*Micromys minutus*) is a small rodent species with a wide range of vertical distribution in Taiwan, extending from the sea level to 3100 m altitude. This species has recently suffered from habitat loss in high-altitude areas due to orchard cultivation, which may have resulted in mouse migration from high to low altitude. To investigate whether there is any physiological mechanism involved in altitude acclimation, rat cDNA microarray was used to compare transcriptomic patterns of the skeletal muscle tissues taken from individuals native to the high-altitude environment and those transferred to the low-altitude captive site. Of the 23,188 genes being analyzed, 47 (33 up-regulated and 14 down-regulated) were found to have differential expression (fold change > 4 or < −4, ANOVA *p* < 0.05). However, after multiple testing correction with a false discovery rate (FDR), only the result for *Tnfrsf12a* was found to be statistically significant (fold change = 13, FDR *p* < 0.05). The result was confirmed by quantitative polymerase chain reaction (q-PCR). The expression of *Tnfrsf12a* possibly relates to the skeletal muscle biology and thus can be correlated with altitude acclimation. However, finding only one gene transcript with significant alteration suggests that transcriptomic response may not play a major role in high- to low-altitude acclimation in harvest mouse.

## 1. Introduction

The harvest mouse (*Micromys minutus*), the sole species of the genus *Micromys*, possesses a vast geographic distribution ranging from the British Isles, northwest Spain through middle Europe to Siberia, Tibet, Assam, and marginal Asian regions including Japan and Taiwan [1,2]. This species is a small rodent with a body size of 5.3 to 6.3 cm which is about half that of the house mouse (*Mus musculus*). Nevertheless, in terms of the phylogenetic relationship, *M. minutus* is more closely related to the Rattini tribe rather than the lineage of *Mus* [3]. In Taiwan, the harvest mouse is often found in habitats at early succession stages, such as grassy fields developed after fire [4]. Among the 13 Taiwanese Murinae species, *M. minutus* has the widest range in vertical distribution, extending from the sea level up to mountains of 3100 m altitude [5]. This species recently suffered habitat loss in high-altitude areas due to orchard cultivation. As a result, population decline was noted in our field surveys in high-altitude habitats. Migration from high to low altitude for species survival was speculated because it has been found that the harvest mouse has a superior migration capacity to cope with natural perturbations, e.g., flooding of habitats [6] and the population distribution of this species is particularly affected by the landscape [7]. It is thus interesting to investigate the physiological adaption and underlying functional genomics regarding altitude change for the harvest mouse.

For endothermic vertebrates inhabiting high-altitude environments, reduced oxygen availability and low ambient temperature are the major environmental stresses. Constant energy production should be maintained while facing hypoxic and colder condition, and certain physiological changes such as increased ventilation rate, increasing cardiac output, increasing red blood cells, and enhanced tissue vascularization can be induced [8,9]. A number of studies have shown that genetic polymorphisms among taxa or populations of high and low altitudes might represent one of the key mechanisms underlying these physiological adaptations [10,11,12]. For example, the regulation of hemoglobin expression is one of the well-known mechanisms underlying adaptation since hemoglobin is responsible for delivering oxygen to tissues, which is vitally important for regulating the metabolism under hypoxic environments [13,14,15]. In addition to the DNA-level genetic divergence, changes of gene expression pattern may also play a role in the physiological adaptation to environmental changes [16]. Particularly in stressful environments, the up- or down-regulation of hormones and proteins are often noted in correlation with increases or decreases in transcription levels [17,18,19,20].

It is also well known that in order to maintain a stable body temperature considerably higher than the outside temperature in high-altitude environments, endothermic animals need effective heat production to counteract the continuous body heat loss. Skeletal muscle activities such as mobility or shivering become a very important mechanism of thermogenesis in mammals, especially at high altitude [21,22]. Meanwhile, structural–functional adjustments of the skeletal muscle were noted in fully altitude-adapted taxa. For example, high-altitude birds have more capillaries and more oxidative locomotive muscles [23,24]. Besides, increased oxidative capacity in the locomotive, respiratory, and cardiac muscles of highland mammals have also been reported [10,25]. These derived physiological changes may increase the capacity for oxygen diffusion and aerobic metabolism in the skeletal muscles and may facilitate thermogenesis under cold and hypoxic environments. Therefore, studies in skeletal muscle activity and the underlying gene regulation are helpful to understand the altitude acclimation of species.

Recent technical advances in DNA sequencing, genotyping and genome-wide expression profiling, coupled with bioinformatics approaches, provide greater feasibility to study the gene expression pattern of the adaptive physiology in animals [19,26,27,28]. Among them, microarray is a powerful and cost-effective tool for the large-scale analysis of gene expressions. It can be used to measure the relative quantities of specific mRNAs in tissue samples for thousands of genes simultaneously [10,29,30,31], therefore allowing for the genome-wide correlation of gene transcript levels with physiological responses and alterations in physiological states during the acclimation of environmental stresses [32,33]. Many metabolism-associated genetic changes in animals have been reported in environments of different altitudes [10,28,34,35].

In this study, we used rat cDNA microarrays to study the variation in transcriptomic patterns of skeletal muscle tissue between two groups of harvest mice. One was sampled from their native high-altitude environment, and the other was sampled from the same population transferred to a captive low-altitude site. We aimed to discover whether there is a transcriptomic response for the high- to low-altitude acclimation in the harvest mouse.

## 2. Materials and Methods

### 2.1. Sample Collection

**Figure 1:**
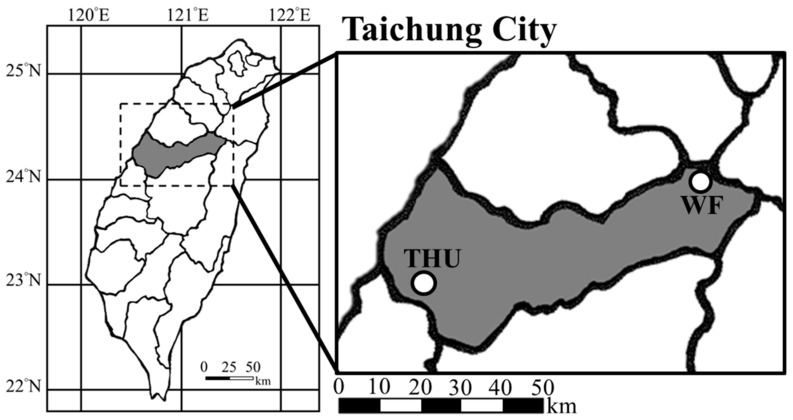
Map showing sampling locations of *Micromys minutus* at Wuling Farm (WF), Taichung, Taiwan (24°24′N, 121°18′E; 2200 m altitude). Some of the captured animals were transferred to and housed in the laboratory of Tunghai University (THU), Taichung, Taiwan (24°18′N, 120°60′E; 200 m altitude) for three weeks before sacrifice.

Eight adult males harvest mice (*M. minutus*) were captured by Sherman live traps from a grassy field of Wuling Farm in central Taiwan, located approximately 2200 m above sea level (24°24′N, 121°18′E). Four of the 8 harvest mice were sacrificed soon after capture. The gastrocnemius muscles were taken from the animals, minced and put in the RNAlater RNA stabilization solution (Thermo Fisher Scientific, Wilmington, DE, USA). The above procedures were performed at the native altitude. The remaining four animals were transferred to the laboratory in Tunghai University, Taichung, Taiwan, located approximately 200 m above sea level (24°18′N, 120°60′E) (Figure 1), and were housed in cages at ambient temperature for three weeks, and then their muscle tissues were sampled using the same procedures mentioned above.

### 2.2. Tissue Preparation and RNA Isolation

Total RNA was extracted from 50 mg of gastrocnemius muscle tissue using TRIzol (Invitrogen, Carlsbad, CA, USA) in conjunction with an RNeasy Mini Kit (Qiagen, Redwood City, CA, USA) according to the manufacturers’ protocols. RNA quality and purity were assessed based on the absorbances at 260 nm and 280 nm using ND-1000 spectrophotometer (Labtech International, Heathfield, East Sussex, UK).

### 2.3. RNA Processing and Microarray Analysis

Approximately 300 ng of total RNA for each sample were preprocessed for hybridization of Rat Clariom S Array (Affymetrix, Santa Clara, CA, USA) using the GeneChip WT PLUS Reagent Kit (Affymetrix, Santa Clara, CA, USA). In brief, the first strand complementary DNA (cDNA) was generated from total RNA using reverse transcriptase and primers containing a T7 promoter sequence. The single-stranded cDNA was then converted to double-stranded cDNA by DNA polymerase and RNase H to simultaneously degrade the RNA. Complementary RNA (cRNA) was synthesized and amplified by in vitro transcription of the second-stranded cDNA template using T7 RNA polymerase. Sense-strand cDNA was then synthesized by the reverse transcription of cRNA with incorporated deoxyuridine triphosphate. Purified, sense-strand cDNA was fragmented by uracil-DNA glycosylase and apurinic/apyrimidinic endonuclease 1 at the unnatural dUTP residues and labeled by terminal deoxynucleotidyl transferase using the Affymetrix proprietary DNA Labeling Reagent. Each labeled sense-strand cDNA fragment was individually hybridized to the array chip at 45 °C for 17 h. Subsequent wash and staining were performed using the Affymetrix GeneChip Wash and Stain Kit, following the manufacturer’s protocol. An image-on-chip was acquired with an Affymetrix GeneChip Scanner 3000 and analyzed with the Transcriptome Analysis Console 2.0 (Affymetrix, Santa Clara, CA, USA).

### 2.4. Statistical Analysis

Lists of apparently differentially expressed genes were generated by finding genes that differed more than 4-fold in expression in the samples grouped by the altitude parameter (low vs. high). Statistically significant differences were determined using one-way analysis of variance (ANOVA, *p* < 0.05), followed by multiple testing correction for a false discovery rate (FDR) [36], with a cut-off of 0.05.

### 2.5. Gene Ontology and Pathway Analyses

The differentially expressed genes were subjected to gene ontology and metabolic pathway analyses using the Database for Annotation, Visualization, and Integrated Discovery (DAVID) (https://david.ncifcrf.gov/) and the separate open source pathway resource of WikiPathways [37] to investigate the biological meaning behind the gene list and extract gene association networks from molecular pathways to predict the biological significance of the gene expression.

### 2.6. Quantitative Real-Time Polymerase Chain Reaction (qPCR) Confirmation

To verify the only gene (*Tnfrsf12a*) with a significant FDR *p* < 0.05 as detected in the aCGH analysis, quantitative reverse transcription PCR (qRT-PCR) was further performed. Two primer sets were used: (1) Tnfrsf12a-qF: 5′-GACCTCGACAAGTGCATGG-3′ and Tnfrsf12a-qR: 5′-CTCGCCACCAGTCTCCTCTA-3′, which are specific for the *Tnfrsf12a* target gene; (2) Hprt1-qF: 5′-GCCAAGGATTTGGAAAAGGT-3′ and Hprt1-qR: 5′-ACAGAGGGCCACAATGTGAT-3′, which are specific for the *Hprt1* as endogenous control [38]; the estimated sizes of the amplified fragments were 228 and 114 base pairs (bp), respectively. The qRT-PCR was performed using the LightCycler 480 II Real-Time PCR System (Roche Applied Science, Mannheim, Germany). The reaction mixture (final volume = 20 µL) contained the cDNA template, 0.4 µM forward and reverse primers, and 1× Smart Quant Green Master Mix (Protech Technology, Taipei, Taiwan). The cycling and melting conditions were as follows: one cycle of 95 °C for 10 min; 45 cycles of 95 °C for 15 s, 60 °C for 1 min. The melting curve analysis was performed at the end of the PCR to check the specific amplification of the target. The amplification profiles were analyzed using the LightCycler 480 Software v1.5.0. The Cq (quantitation cycle) values were determined by auto-baseline correction, setting the threshold to lie within the exponential phase of all amplification curves and exporting data into a Microsoft Excel worksheet for analysis. Relative expression of the target gene was determined by a ΔΔCq calculation method.

## 3. Results

### 3.1. High- to Low-Altitude Transfer

All four harvest mice were captured in a high-altitude area (2200 m altitude) and subsequently transferred to the low-altitude laboratory (200 m altitude) and housed in cages for three weeks. They were healthy, fed normally, and without any notable injury before being sacrificed.

### 3.2. Microarray Analysis

Of the 23,188 gene transcripts analyzed in the Rat Clariom S Array (Affymetrix), 47 (0.20%) showing differential expression patterns (33 or 70.21% were up-regulated and 14 or 29.79% were down-regulated) between the high-altitude and low-altitude harvest mouse groups were identified with a cut-off fold change > 4 or < −4, and *p* < 0.05 (Table 1 and Table S1). Of the 47 genes with differential expression, 17 were indicated by WikiPathways to involve in 31 different biological pathways (Table 2). After filtering with FDR multiple testing correction, only one gene *Tnfrsf12a* showed statistical significance. The expression of *Tnfrsf12a* increased 13-fold in the low-altitude environment when compared with that in the high-altitude environment (Table S1).

### 3.3. qPCR Analysis

The up-regulated expression of *Tnfrsf12a* in the low-altitude environment was further confirmed by qPCR (increased 7.85 ± 1.32 fold) (Table 3).

## 4. Discussion

The harvest mouse prefers grasslands with large patches sizes and perennial plant types [39], mostly because their nesting is influenced by the height of vegetation [40]. Due to grass mowing for many reasons, such as riverbank management or agricultural practices, this species is endangered by grassland habitat loss worldwide [41]. Due to its wide vertical distribution in Taiwan, it is interesting to elucidate the mechanisms underlying their great adaptation capability. In this study, we used microarray analysis to obtain a comprehensive understanding of the gene expression differences of the harvest mouse between environments of different altitudes. We sought to understand how the harvest mouse adapts to such environmental stress from the transcriptomic perspective when a high- to low-altitude acclimation was speculated. We found that only the *Tnfrsf12a* gene showed a significant transcriptomic response using the rat cDNA microarray, and the result was confirmed by qPCR.

The *Tnfrsf12a* gene encodes a cell membranous protein Tnfrsf12a that is referred to in the literature as either fibroblast growth factor-inducible 14 (Fn14) or a TWEAK receptor (TweakR). Tnfrsf12a is recognized as a TNF receptor family member able to transduce TWEAK signals [42]. Low expression of Tnfrsf12a was reported for cells of healthy homeostatic tissue, and high Tnfrsf12a expression was regularly found on the cells of tissue with damage and regeneration [43]. A recent study demonstrated that the TWEAK–Tnfrsf12a system plays a crucial role in skeletal muscle biology [44]. Skeletal muscle exhibits strong regenerative capacity, as a means to recover from injury as well as to adapt to changing physical demands [45]. It has been showed that the activity of the TWEAK–Tnfrsf12a is greatly dependent on the induction of Fn14 expression [46]. Tnfrsf12a protein expression will be induced after exercise [47,48]. In our study of the muscle tissue of the harvest mice, *Tnfrsf12a* was more expressed in the low-altitude environment. Such finding may be explained by the higher demand for exercise and higher turnover of the skeletal muscle cells in the low-altitude group animals. However, the possibility that the increase of *Tnfrsf12a* expression may have been caused by an unrecognizable injury in animals due to the captive environment cannot be excluded.

Similar studies of other species regarding altitude acclimation took the skeletal muscle as the specimen mainly because of its importance in energy consumption and heat production for endothermal animals [10,20,22,23,24]. Nevertheless, in this harvest mouse study, a significant change of gene expression profiles in different altitudes was not noted. A possible explanation for this is that other organ systems, such as the hematologic or circulatory system, may play more important roles than the skeletal muscle with regard to environmental changes [49]. Another factor that should be considered is that the magnitude of altitude changes adopted in this study was not sufficiently large. Human studies have showed that the size of muscle fibers does not change up to an altitude of 3500 m, whereas above this level, the higher the altitude, the sooner and more apparent hypotrophy was noted [50]. It has been shown that the muscle size of the leg was reduced by 10–15% in sea-level residents who lived at 3700 m above sea level for 32 weeks and were reasonably physically active [51], and the size of the muscle fibers of people who remained at 4100 m above sea level for eight weeks decreased by 8% [52]. Another human study showed that both muscle protein degradation and synthesis rates were unaffected by hypoxia at 4500 m above sea level [53]. These studies demonstrated that the muscle can adapt to altitude changes, but the extent to which the hypoxia plays the major role is still uncertain. In our study, the altitude difference between our study sites was only about 2000 m (i.e., 2200–200 m). The alteration at altitude in this study may be minor for the muscle cells.

Besides the physiological changes, the behavior of the harvest mouse may also play a role in the altitude acclimation. The harvest mouse does not hibernate and aestivate when environmental stresses (e.g., low or high temperature) are encountered [1]. In contrast, the harvest mouse builds special globular nests (about 5–9 cm in diameter) composed of three or four layers, each handled in a different manner [1]. The nest can be suspended 10–200 cm above the ground or built on the ground or under the surface litter layer [54]. Such nests have good insulating properties [55,56]. It has been suggested that *M. minutus* has nest-sharing nesting behavior, huddling in a single bottom nest, constructed by not only breeding females but also males and non-breeding females [54]. The harvest mouse cannot dig, but some bottom nests were found connected to underground burrows of more than 20 cm in length [55]. This behavior may help the harvest mouse escape from fire or snow. With such warm nests and various adaptive behaviors, the body heat and metabolism of the harvest mouse may be maintained without significant physiological changes. This may provide a possible explanation for our finding that there was no significant different gene expression profile between the harvest mice living at different altitudes.

However, it should be noted that the above results were found solely in the skeletal muscle tissue, which may directly or indirectly reflect physiological changes in the animals. Meanwhile, moving the animals from their native high-altitude environment to the low-altitude captive environment may not truly reflect the population migration and thus the altitude acclimation in natural habitats.

## 5. Conclusions

In summary, only one gene was identified to be significantly differentially expressed between the muscle tissues of harvest mice in environments of different altitudes. This finding implies that transcriptomic responses may not play a major role in the altitude acclimation of the harvest mouse group we studied. Future studies of comparative functional genomics involving other organ systems (in addition to skeletal muscles) and a larger sample size are warranted for a better understanding of how this small rodent species *M. minutus* can possess such wide distribution ranges both in both the horizontal and vertical dimensions.

## Figures and Tables

**Table 1:** Summary of differentially-expressed patterns between harvest mice from the high-altitude environment (H) and then acclimated to the low-altitude environment (L), as detected by Affymetrix Rat Clariom S Array with the filter criteria of L/H fold change > 4 or < −4, and ANOVA *p* < 0.05. Number of filtered genes with an FDR *p* < 0.05 after a multiple testing correction was also shown (for details, please see Table S1).

No. of Total Genes Examined	23,188
No. of filtered genes (% of examined genes)	47 (0.20%)
No. of up-regulated genes (% of filtered genes)	33 (70.21%)
No. of down-regulated genes (% of filtered genes)	14 (29.79%)
No. of filtered genes with FDR *p* < 0.05 (% of filtered genes)	1 (2.13%)

**Table 2:** List of differentially-expressed genes and biological pathways between harvest mice from the high-altitude environment (H) and then acclimated to the low-altitude environment (L) (i.e., L/H) with a cut-off fold change > 4 or < −4, and ANOVA *p* < 0.05, assessed by WikiPathways [37].

Pathway	No. of Differentially Expressed Genes	Up-Regulated Genes		Down-Regulated Genes	
		Number	List	Number	List
CDKN1A-EGF-CREB	3	3	*Hbb, Cdkn1a, Hbb-b1*	0	
IL-2 Signaling Pathway	2	2	*Il2rg, Cd53*	0	
MAPK Signaling Pathway	2	0		2	*Nr4a1, Cacng6*
B Cell Receptor Signaling Pathway	2	2	*Blnk, Cd79a*	0	
Tryptophan metabolism	2	1	*Cyp2f4*	1	*Cyp2e1*
Nuclear factor, erythroid-derived 2, like 2 signaling pathway	2	1	*Cdkn1a*	1	*Cacng6*
Heme Biosynthesis	1	1	*Alas2*	0	
IL-9 Signaling Pathway	1	1	*Il2rg*	0	
ATM Signaling Pathway	1	1	*Cdkn1a*	0	
Electron Transport Chain	1	1	*Ucp2*	0	
Alpha6-Beta4 Integrin Signaling Pathway	1	1	*Cdkn1a*	0	
Hypertrophy Model	1	0		1	*Dusp14l1*
Cell cycle	1	1	*Cdkn1a*	0	
Ethanol metabolism resulting in the production of ROS by CYP2E1	1	0		1	*Cyp2e1*
Inflammatory Response Pathway	1	1	*Il2rg*	0	
TGF-beta Receptor Signaling Pathway	1	1	*Cdkn1a*	0	
G1 to S cell cycle control	1	1	*Cdkn1a*	0	
Cytoplasmic Ribosomal Proteins	1	1	*Rpl35*	0	
Relationship between glutathione and NADPH	1	1	*Cdkn1a*	0	
Spinal Cord Injury	1	0		1	*Nr4a1*
Nuclear Receptors	1	0		1	*Nr4a1*
Serotonin and anxiety	1	1	*Plek*	0	
IL-4 Signaling Pathway	1	1	*Il2rg*	0	
Adipogenesis	1	1	*Cdkn1a*	0	
Nuclear receptors in lipid metabolism and toxicity	1	0		1	*Cyp2e1*
Fatty Acid Omega Oxidation	1	0		1	*Cyp2e1*
Senescence and Autophagy	1	1	*Cdkn1a*	0	
ErbB signaling pathway	1	1	*Cdkn1a*	0	
Retinol metabolism	1	0		1	*Cyp2e1*
Metapathway biotransformation	1	0		1	*Cyp2e1*
IL-7 Signaling Pathway	1	1	*Il2rg*	0	

**Table 3:** Fold change of *Tnfrsf12a* expression between harvest mice from the high-altitude environment (H) and then acclimated to the low-altitude environment (L), calculated by ΔΔCq method. Data are expressed as mean ± standard deviation.

Sample	*Tnfrsf12a* (Average Cq)	*Hprt1* (Average Cq)	ΔCq (*Tnfrsf12a* − *Hprt1*)	ΔΔCq (ΔCq _Low altitude_ − ΔCq _High altitude_)	Fold Change (2^−(ΔΔCq)^)
High altitude (*n* = 4)	28.46 ± 1.36	22.40 ± 1.24	6.05 ± 0.25	-	-
Low altitude (*n* = 4)	26.73 ± 2.71	23.63 ± 2.68	3.10 ± 0.11	−2.95 ± 0.25	7.85 ± 1.32

-: Not calculated.

## Supplementary Materials

The following are available online at https://www.mdpi.com/2076-2615/9/3/92/s1, Table S1: Details of the 47 differentially expressed genes between harvest mice from a high-altitude environment (H) and acclimated to a low-altitude environment (L), as detected respectively by Affymetrix Rat Clariom S Array with filter criteria of fold change of L/H > 4 or < -4, and ANOVA *p* < 0.05. The filtered genes were sorted in ascending order based on the FDR *p*-value [36]. The only one gene (*Tnfrsf12a*) with FDR *p* < 0.05 was marked in bold.
