# Supplementary material for: Genome-Wide Microarray Analysis Suggests Transcriptomic Response May Not Play a Major Role in High- to Low-Altitude Acclimation in Harvest Mouse (Micromys minutus)
Source: Animals (Basel). 2019 Mar 13;9(3):92. doi: 10.3390/ani9030092 (PMC6466072; doi:10.3390/ani9030092)
Supplement: Supplementary file 1 [file animals-09-00092-s001.pdf]

Table S1. Details of the 47 differentially expressed genes between harvest mice from a high-altitude environment (H) and acclimated to a low-altitude environment (L), as detected respectively by Affymetrix Rat Clariom S Array with filter criteria of fold change of L/H > 4 or < -4, and ANOVA $p < 0.05$. The filtered genes were sorted in ascending order based on the FDR p-value [36]. The only one gene (*Tnfrsf12a*) with FDR $p < 0.05$ was marked in bold.

Gene	ID	Fold Change	p	FDR p	Description
Tnfrsf12a	**TC1000002359.rn.2**	**13.0**	**1.06E-06**	**0.0246**	**Tumor necrosis factor receptor superfamily, member 12a**
Arhgap20	TC0800000694.rn.2	4.6	0.0005	0.3997	Rho GTPase activating protein 20
Cd53	TC0200004423.rn.2	7.3	0.0007	0.3997	Cd53 molecule; INVOLVED IN positive regulation of myoblast fusion (ortholog) AND FOUND IN cell surface AND cell-cell junction (ortholog) AND extracellular vesicular exosome (ortholog) AND INTERACTS WITH 17alpha-ethynylestradiol AND 2,3,7,8-tetrachlorodibenzodioxine AND 3H-1,2-dithiole-3-thione
Ucp2	TC0100002142.rn.2	5.0	0.0008	0.3997	Uncoupling protein 2 (mitochondrial, proton carrier)
Slc25a25	TC0300002876.rn.2	-25.6	0.001	0.4313	Solute carrier family 25 (mitochondrial carrier, phosphate carrier), member 25
Ankrd23	TC0900001821.rn.2	-4.4	0.0011	0.4328	Ankyrin repeat domain 23
Hbb	TC0100006234.rn.2	14.5	0.0014	0.4659	Hemoglobin, beta
RGD1565183	TC0X00002652.rn.2	6.5	0.0017	0.4659	ENCODES a protein that exhibits structural constituent of ribosome (inferred) AND INVOLVED IN translation (inferred) AND FOUND IN ribosome (inferred) AND INTERACTS WITH C60 fullerene
Slc7a2	TC1600001707.rn.2	-4.2	0.0021	0.4659	Solute carrier family 7 (cationic amino acid transporter, y+ system), member 2
Cdkn1a	TC2000000249.rn.2	4.9	0.0023	0.4659	Cyclin-dependent kinase inhibitor 1A
Dusp14l1	TC0200001120.rn.2	-4.1	0.0027	0.4659	Dual specificity phosphatase 14-like 1 [Source: RGD Symbol; Acc: 1590821]; ENCODES a protein that exhibits MAP kinase tyrosine/serine/threonine phosphatase

					activity (inferred) AND<br>protein tyrosine phosphatase activity (inferred) AND<br>INVOLVED IN inactivation of MAPK activity (inferred) AND<br>INTERACTS WITH (S)-colchicine (ortholog) AND<br>3-methylcholanthrene (ortholog) AND<br>5-fluorouracil (ortholog)
Hbb-b1	TSUnmapped00000112.rn.2	4.2	0.0028	0.4659	Hemoglobin, beta adult major chain
Cacng6	TC0100004649.rn.2	-4.7	0.0031	0.4659	Calcium channel, voltage-dependent, gamma subunit 6
Rpl35	TC0300003018.rn.2	4.4	0.0037	0.4845	Ribosomal protein L35
LOC100134871	TC0100002239.rn.2	12.1	0.0037	0.4845	Beta globin minor gene
LOC100361705	TC0300000347.rn.2	4.7	0.0046	0.4845	rCG64259-like [Source: RGD Symbol; Acc: 2319569]
Arhgap15	TC0300000497.rn.2	6.8	0.0061	0.4924	Rho GTPase activating protein 15
Ighg; Igh-6	TC0600003269.rn.2	6.1	0.0069	0.5032	Immunoglobulin heavy chain (gamma polypeptide); immunoglobulin heavy chain 6
Plek	TC1400002279.rn.2	4.8	0.0072	0.5032	Pleckstrin
Ikzf1	TC1400001003.rn.2	7.3	0.0083	0.5065	IKAROS family zinc finger 1
Cyp2f4	TC0100001106.rn.2	7.8	0.0087	0.5082	Cytochrome P450, family 2, subfamily f, polypeptide 4
Hbb-b1; LOC689064	TCUn_AABR07024041v100000002.rn.2	20.2	0.0091	0.5084	Hemoglobin, beta adult major chain [Source: RGD Symbol; Acc: 1595848]; beta-globin hemoglobin, beta adult major chain; ENCODES a protein that exhibits heme binding (inferred) AND<br>iron ion binding (inferred) AND<br>oxygen binding (inferred) AND<br>INVOLVED IN oxygen transport (inferred) AND<br>PARTICIPATES IN Plasmodium infection pathway AND<br>*Trypanosoma brucei* infection pathway AND<br>FOUND IN hemoglobin complex (inferred) AND<br>INTERACTS WITH (-)-demecolcine (ortholog) AND<br>17beta-estradiol (ortholog) AND<br>2,2',4,4',5,5'-hexachlorobiphenyl (ortholog); hemoglobin,

					beta adult major chain (Hbb-b1), mRNA.
Rgs13	TC1300001554.rn.2	4.2	0.0109	0.5084	Regulator of G-protein signaling 13 [Source: RGD Symbol; Acc: 1562103]; INVOLVED IN G-protein coupled receptor signaling pathway (ortholog) AND negative regulation of G-protein coupled receptor protein signaling pathway (ortholog) AND FOUND IN cytosol (ortholog) AND nucleus (ortholog) AND plasma membrane (ortholog) AND INTERACTS WITH lead diacetate AND 17beta-estradiol (ortholog) AND 5-fluorouracil (ortholog)
Laptm5	TC0500001570.rn.2	4.3	0.0115	0.5084	Lysosomal protein transmembrane 5
Blnk	TC0100007686.rn.2	6.3	0.0137	0.5187	B-cell linker; ENCODES a protein that exhibits SH3/SH2 adaptor activity (ortholog) AND INVOLVED IN intracellular signal transduction (ortholog) AND positive regulation of signal transduction (ortholog) AND PARTICIPATES IN phosphatidylinositol 3-kinase class I signaling pathway AND B cell receptor signaling pathway AND primary immunodeficiency disease pathway AND ASSOCIATED WITH Agammaglobulinemia (ortholog) AND AGAMMAGLOBULINEMIA 4 AUTOSOMAL RECESSIVE (ortholog) AND FOUND IN cytoplasm (ortholog) AND INTERACTS WITH beta-naphthoflavone AND diuron AND indole-3-methanol
Tcrb	TC0400000725.rn.2	61.2	0.0144	0.5198	T-cell receptor beta chain
Nr4a1	TC0700001870.rn.2	-4.1	0.0156	0.5268	nuclear receptor subfamily 4, group A, member 1
Il2rg	TC0X00001947.rn.2	50.0	0.0156	0.5268	interleukin 2 receptor, gamma

Alas2	TC0X00001581.rn.2	52.2	0.0158	0.5268	5-aminolevulinate synthase 2
Dnase1l3	TC1500002466.rn.2	4.8	0.0171	0.5276	ENCODES a protein that exhibits DNA binding AND endodeoxyribonuclease activity AND endonuclease activity (ortholog) AND INVOLVED IN apoptotic DNA fragmentation (ortholog) AND developmentally programmed cell death (ortholog) AND FOUND IN nucleus AND INTERACTS WITH 3-chloropropane-1,2-diol AND ammonium chloride AND cadmium dichloride
Hmgb2l1	TC1100001483.rn.2	8.9	0.0183	0.5333	high mobility group box 2-like 1
Dnase1l3	TC1500002467.rn.2	4.8	0.019	0.5374	deoxyribonuclease 1-like 3
Myom2	TC1600001984.rn.2	-4.0	0.0209	0.5451	myomesin 2
Hist1h2bcl1	TC1700001825.rn.2	4.9	0.022	0.5477	Histone H2B; ENCODES a protein that exhibits DNA binding (inferred) AND protein heterodimerization activity (inferred) AND INVOLVED IN nucleosome assembly (inferred) AND PARTICIPATES IN systemic lupus erythematosus pathway AND FOUND IN nucleosome (inferred) AND nucleus (inferred) AND INTERACTS WITH (-)-demecolcine (ortholog) AND (S)-nicotine (ortholog) AND 2,3,7,8-tetrachlorodibenzodioxin (ortholog)
Nnat	TC0300002176.rn.2	-318.5	0.0224	0.5477	Neuronatin
Cd79a	TC0100001061.rn.2	5.1	0.0251	0.5598	Cd79a molecule, immunoglobulin-associated alpha
Smco1	TC1100001758.rn.2	-4.6	0.0261	0.5608	Single-pass membrane protein with coiled-coil domains 1
Tcrb	TC0400000714.rn.2	4.3	0.0275	0.5659	T-cell receptor beta chain
Hist2h3c2	TC0200001754.rn.2	4.8	0.0276	0.5659	Histone cluster 2, H3c2
LOC100911042	TC1100001759.rn.2	-4.8	0.0324	0.5702	Uncharacterized LOC100911042
Alb	TC1400001516.rn.2	-26.8	0.0329	0.573	Albumin

Rbm3	TC0X00000125.rn.2	-4.9	0.0329	0.573	RNA binding motif (RNP1, RRM) protein 3
Ms4a6b	TC0100003231.rn.2	5.4	0.0345	0.5816	Membrane-spanning 4-domains, subfamily A, member 6B
LOC100360581	TC0600003308.rn.2	5.1	0.0377	0.5826	Uncharacterized protein; rCG58847-like
Neurl1	TC0100003765.rn.2	-4.9	0.04	0.5826	Neuralized E3 ubiquitin protein ligase 1
Tmsb10; LOC100364435	TC0700001491.rn.2	4.2	0.0432	0.5905	Thymosin, beta 10 [Source: RGD Symbol; Acc: 62022]; thymosin beta 10-like
Cyp2e1	TC0100002862.rn.2	-9.2	0.0433	0.5905	Cytochrome P450, family 2, subfamily e, polypeptide 1
